# Factors Affecting Nurses' Walking Distance: Age, Clinical Ladder Level, Wards, Nurse Calls, Weekend, and Patient-To-Nurse Ratio

**DOI:** 10.1155/jonm/5540600

**Published:** 2025-08-28

**Authors:** Keisuke Nakashima, Yoshiaki Inoue, Tomoyuki Iwasaki, Haruna Fukushige, Atsue Ishii

**Affiliations:** ^1^Department of Medical Informatics, Graduate School of Medicine, The University of Osaka, 2-2 Yamadaoka, Suita, Osaka 565-0871, Japan; ^2^Department of Information and Communications Technology, Graduate School of Engineering, The University of Osaka, 2-1 Yamadaoka, Suita, Osaka 565-0871, Japan; ^3^Division of Nursing, The University of Osaka Hospital, 2-15 Yamadaoka, Suita, Osaka 565-0871, Japan; ^4^Department of Nursing, Graduate School of Health Sciences, Kobe University, 7-10-2 Tomogaoka, Suma-ku, Kobe, Hyogo 654-0142, Japan; ^5^Division of Health Science, Graduate School of Medicine, The University of Osaka, 1-7 Yamadaoka, Suita, Osaka 565-0871, Japan

## Abstract

In many developed countries, a rapidly aging population has increased healthcare demands and the proportion of older nurses in the workforce. This demographic shift requires nursing managers to have a deeper understanding of the physical demands on staff nurses, particularly older ones. In this paper, we aim to provide valuable insights for developing evidence-based strategies to improve work environments in hospital wards. To achieve this, we conducted a statistical analysis of data on walking distance for day, long-day, and night shifts, obtained from a long-term survey across 14 wards in a large acute care hospital in Japan using automated data collection via mobile devices. Using nonparametric multiple comparisons and multiple regression analysis, we evaluated the impact of factors such as age, clinical ladder level, years of service, ward type, nurse calls, weekend, and patient-to-nurse ratio on walking distance. The multiple comparison tests revealed significant differences in walking distance among clinical ladder levels, with small to medium effect sizes. While age and years of service had some impact, their influence was less pronounced than that of clinical ladder levels. Our regression analysis showed that ward characteristics significantly affected walking distance, with emergency wards exhibiting notably longer distances. The number of nurse calls had a significant positive impact on walking distance across all shifts, while the patient-to-nurse ratio significantly affected walking distance only for night shifts. The weekend affected walking distance only for long-day shifts. These findings suggest that ward managers should reexamine the appropriate nursing care systems suited to the characteristics of their ward, and that reconsidering approaches to task assistance for less experienced nurses and night shift allocations for novice nurses could effectively reduce physical burdens on nurses. They also emphasize the importance of workload balancing in task and patient assignments and the consideration of ward characteristics in nurse reshuffling.

## 1. Introduction

**Figure 1:**
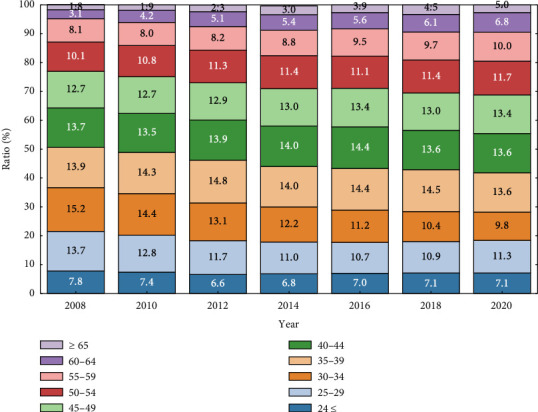
Transition of nursing staff age groups in Japan.

In many developed countries, declining birth rates and an aging population, coupled with advances in medical care, have led to an increased demand for nursing care and a shortage of nurses. This situation has resulted in higher workloads and heavier physical burdens on nurses. Moreover, the aging population affects not only the proportion of older patients but also that of older nurses.  Figure 1 shows the transition of nursing staff age groups from 2008 to 2020 in Japan [1]. It indicates a clear increase in the proportion of those aged 50 and above, with a 10.4% increase from 2008 to 2020. Furthermore, Japan is making ongoing efforts to support the continued employment of nurses who work past retirement age. Thus, it is reasonable to expect a further increase in the proportion of older nurses in the future.

Previous studies have reported the impact of high physical demand tasks [2], musculoskeletal problems [3, 4], and stress [5] on nurses' health, as well as the impact of the work environment on turnover rates [6, 7] and job satisfaction [8]. In addition, nurses' health and turnover rates are adversely affected by shift work [9] and an increase in workload [10], emphasizing the need to improve work environments to maintain nurses' health [11]. Furthermore, older nurses are less likely to achieve the recommended levels of physical activity at work, making it crucial to develop strategies such as assigning tasks suitable for their capabilities [12]. Considering these studies and the increase in the proportion of older nurses, Japanese hospitals urgently need to develop strategies to improve their work environments and enable older nurses to continue working as long as possible.

To develop such strategies, it is important to accurately understand the actual nursing workload and workflow, which can vary daily due to various factors such as patient severity, bed utilization rates, surgeries, and tests. This understanding requires analyzing large amounts of data from long-term surveys. However, conventional time and motion study methods, that is, self-recording and third-party recording, which have been widely used in many surveys [13–18], are not suitable for long-term surveys due to their cost and labor intensity. These conventional methods are inherently resource-intensive. Self-recording obliges the on-duty nurse to pause clinical work every three to five minutes to log activities, imposing a substantial cognitive and physical burden. Third-party recording, meanwhile, requires one trained observer for each nurse throughout all scheduled working hours, including night shifts, which significantly increases personnel costs in a 24-h ward.

Recently, advances in Internet-of-Things (IoT) technologies have led to the development of unmanned time study methods using RFID tags, beacons, and mobile devices, which are gaining attention as new methods for conducting long-term surveys [19–21]. These methods automatically collect objective data for analyzing nurses' workloads and movement lines, thus reducing both financial costs and the burden on participants. Among the various metrics captured by these systems, walking distance has been shown to serve as a valid proxy for physical burden. For example, Chang and Cho [22] reported that objectively measured steps and walking distance closely mirrored nurses' perceived physical demand scores across day, evening, and night shifts, suggesting that cumulative walking distance reflects the shift-level workload as subjectively experienced by nurses. In this study, we used data from a long-term survey employing unmanned time study methods with beacons and mobile devices [21], which was conducted across 14 wards in a large acute care hospital in western Japan. Although this survey collected data on nurses' walking distance and the beacons' received signal strength indicators (RSSIs), we focus on nurses' walking distance in this paper as a physiologically grounded indicator of physical demand.

There are several related studies on nurses' walking distance [19, 22–26]. Welton et al. conducted a survey in four units of a large university hospital and showed that the average walking distance for 12-h day and night shifts was approximately 6.56 and 6.4 kilometers [23]. They also examined the impact of the number of assigned patients on walking distance, but their findings were limited by the small sample size. Hendrich et al. conducted time and motion studies using RFID tags and mobile devices in 36 hospitals and reported that median walking distance per 10 h for day and night shifts was 4.8 and 3.5 kilometers [19]. They also analyzed the time nurses spent on various tasks and locations. While these studies provided statistical data on nurses' walking distance, factors influencing walking distance were not discussed. On the other hand, Chang and Cho conducted a survey in two tertiary hospitals with a three-shift system and evaluated the impact of nurses' educational levels, unit types, ages, and years of ward experience on walking distance by regression analysis, identifying unit types and years of ward experience as significant factors [22]. They also showed that nurses aged under 28 tended to walk more than those aged 28 and above. However, their participants were confined to young nurses around 30 years old. Other studies [24–26] have examined the impact of ward layout on walking distance.

In this paper, we identify factors affecting nurses' walking distance using data from the 14-ward survey, where participants (nurses) ranged in age from 22 to 60 years old. We conduct multiple comparisons to evaluate differences among groups based on clinical ladder level (a structured indicator of nursing skill level), age, and years of service, where clinical ladder level and years of service are not discussed in previous studies [22, 23]. In clinical settings, practical experience suggests that clinical ladder levels and years of service affect both movement patterns and total walking distance. For example, nurses with higher clinical ladder levels are more likely to be assigned coordinating or supervisory roles rather than direct patient care, which reduces the frequency of room-to-room movement. In addition, nurses with more years of service often develop better task planning and spatial familiarity, which may reduce unnecessary walking such as returning to the nurse station to retrieve forgotten supplies or making redundant movements due to inefficient task sequencing. Therefore, while clinical ladder level and years of service are interrelated, they may influence walking behavior through distinct pathways. However, these practical assumptions have not been systematically verified through quantitative data. Thus, we aim to fill this gap by analyzing walking distance in relation to clinical ladder levels and years of service using a large-scale dataset collected from 14 wards.

Using multiple regression analysis, we evaluate the relative influence of various factors such as clinical ladder levels, age, ward types, nurse calls, weekend, and patient-to-nurse ratio (PNR) on walking distance. Although previous studies [22, 23] have investigated some of these factors individually, they have not examined them collectively and systematically using a large and heterogeneous sample. In particular, clinical ladder levels, nurse calls, weekend, and PNR have not been analyzed simultaneously in prior research involving diverse nursing units and a wide range of nurse demographics.

Finally, based on the findings from these analyses, we discuss evidence-based strategies to improve the work environment and reduce the physical burden on nurses. As shown in Figure 1, the age distribution of nursing staff in Japan has shifted markedly over the past two decades, with a growing proportion of nurses aged 50 or older. This demographic trend reflects Japan's status as one of the most rapidly aging countries in the world, where both patient and nurse populations are aging in parallel. As a result, Japanese tertiary hospitals offer a unique early model of age-related workload intensification. Studying workload determinants in this context provides valuable insights for other countries facing similar demographic transitions in the near future.

Based on this context, this study has the following objectives: (i) to compare walking distance across nurse groups categorized by clinical ladder level, age, and years of service; (ii) to identify and quantify the relative influence of individual and contextual factors such as clinical ladder levels, ward types, nurse calls, weekends, and PNR on walking distance using regression analysis. These objectives align with the statistical analyses presented in later sections and aim to provide actionable evidence for improving nursing workload management in aging and high-acuity hospital settings.

## 2. Method

### 2.1. Research Design

We conducted a retrospective observational study using data collected from a survey across 14 wards of a large acute care hospital affiliated with a national university in western Japan.

### 2.2. Data Collection and Processing

**Figure 2:**
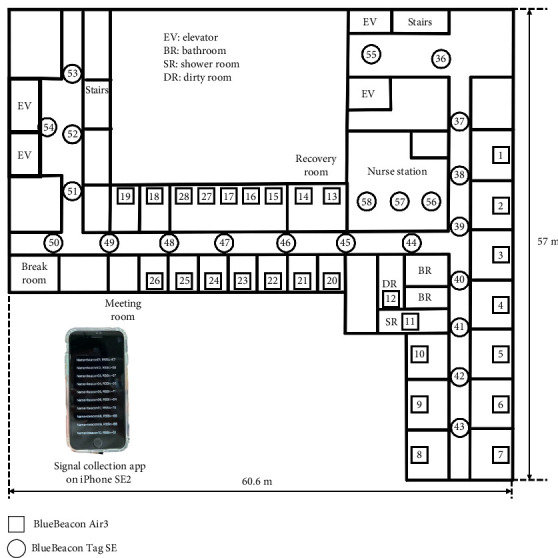
Example of beacon setting.

Data were collected using an unmanned time study method with beacons and mobile devices [21]. As shown in Figure 2, approximately 50 beacons were installed in each target ward. The exact number varied depending on the ward's layout and size, and the figure reflects a typical installation. Participants carried mobile devices (iPhone SE2, Apple Inc.) with a dedicated application running, which recorded the beacon IDs, RSSI values, and timestamps per second while the mobile device was in use. Walking distance data were collected by the preinstalled iOS application called “Health,” which calculates the distance per hour [27]. We extracted the data on walking distance per hour from the mobile devices and calculated the total distance per shift, including break periods. Participants were instructed to carry the mobile device at all times during their shifts to avoid missing data due to forgetting the device. As a result, walking distance measurements included movements during scheduled breaks, such as walking to staff rooms or restrooms.

During the study period, all 14 wards whose head nurses consented to participation were included in the analysis. No wards conducting the same experiment were excluded after data collection. Here, we show an overview of the 14-ward survey in Table 1. Note that while the ward layout, except for ward A, is almost the same as shown in Figure 2, the layout of ward A is unique because of its emergency care function, with computed tomography (CT) and magnetic resonance imaging (MRI) rooms, and the storage of supplies and equipment located far from the nurse station.

#### 2.2.1. Shift System and Participants

The target hospital employs a two-shift system, where the day shift includes an 8-h day shift and a 12-h day (long-day) shift, and the night shift is 12 h long. Typically, nurses who work the long-day shift are also assigned to the night shift on the following day, resulting in equal numbers of nurses on both the long-day and night shifts. The day shift ends at 5:00 p.m., leaving only nurses on the long-day shift after that time until 8:00 p.m., when the night shift starts. Therefore, the number of nurses after 5:00 p.m. is less than that of nurses during the daytime.

Participants were all nurses working on the day, long-day, and night shifts. Although data from late shifts (e.g., 12:00 p.m.–9:00 p.m.) were also collected, these records were excluded because only 22 eligible participants were available, which was an insufficient number for meaningful statistical analysis. Additionally, part-time nurses, ward managers, and leader nurses who primarily perform administrative duties and do not take charge of patients were also excluded. The number of unique participants on the day, long-day, and night shifts was 343, 393, and 393, respectively. No participants were excluded from the analysis due to voluntary withdrawal. As each participant worked several times on each shift during the survey period, the total number of data records for day, long-day, and night shifts was 883, 991, and 1050, respectively, excluding records partially lost due to mobile device battery exhaustion. No records were excluded due to sensor malfunction.

Across all three shifts, no single ward contributed more than 11% of the total records for that shift, indicating that no ward disproportionately influenced the results (see Appendix A for details). Moreover, to confirm that repeated observations were reasonably balanced across nurses, we analyzed the distribution of records per nurse by shift type. Most nurses contributed between one and five observations per shift, and no nurse disproportionately influenced the dataset (see Appendix B for details).

#### 2.2.2. Variables for Analysis

We collected data on nurses' personal information: age, gender, ward, years of service, and clinical ladder level, as well as ward-specific data: occupancy rate, bed utilization rate, number of nurses per shift, number of nurse calls, and weekends/holidays. The clinical ladder is an evaluation system for nursing practice skills. Although the details of the evaluation criteria vary from hospital to hospital, the basic concept of the clinical ladder is based on Benner's model [28], where nursing practice skill levels are divided into five levels: novice, advanced beginner, competent, proficient, and expert. The clinical ladder levels of the target hospital, originally categorized into five levels according to Benner's model, were simplified for this study into four: 0, I, II, and III. We summarized the key criteria for each level in Table 2.

**Figure 3:**
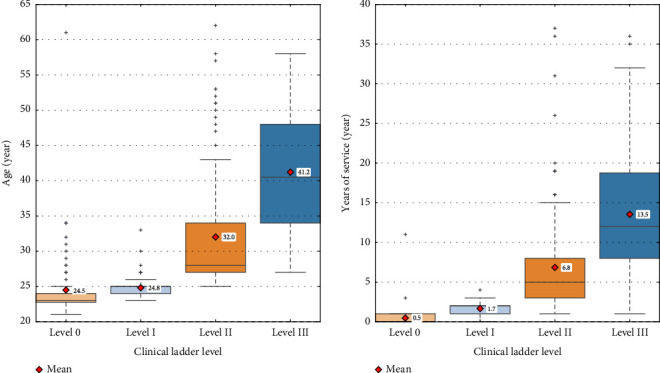
Box plots of age and years of service by clinical ladder level.

The categorization of age and years of service used for multiple comparisons was determined based on a combination of three factors: (i) distinguishing rookie nurses (i.e., less than one year of service), (ii) the distributions of age and years of service by clinical ladder level shown in Figure 3, and (iii) the need to ensure sufficient sample sizes within each group for meaningful statistical comparisons. Consequently, age was categorized into four groups: 24 and under, 25 to 29, 30s, and 40 and over. Years of service was categorized into five groups: zero years (i.e., less than one year), one to two years, three to six years, seven to 12 years, and 13 and over years.

For regression analysis, we calculated the PNR, which is the average number of assigned patients per nurse, in addition to the occupancy rate and bed utilization rate. Let *N*_inp_, *N*_adm_, *N*_dsc_, *N*_tfi_, *N*_tfo_, *N*_ns_^(d)^, *N*_ns_^(n)^, and *N*_b_ denote the numbers of inpatients yesterday, admitted, discharged, transferred in, transferred out, nurses during daytime, nurses during nighttime, and beds, respectively. The occupancy rate is calculated by (*N*_inp_ + *N*_adm_ + *N*_tfi_)/*N*_b_, and the bed utilization rate is calculated by (*N*_inp_ + *N*_adm_ − *N*_dsc_ + *N*_tfi_ − *N*_tfo_)/*N*_b_. The PNR is calculated by (*N*_inp_ + *N*_adm_ + *N*_tfi_)/*N*_ns_^(d)^ for day and long-day shifts and (*N*_inp_ + *N*_adm_ − *N*_dsc_ + *N*_tfi_ − *N*_tfo_)/*N*_ns_^(n)^ for night shifts.

The nurse call system allows patients to call nurses via buttons attached to beds or sensors built into beds and mats. Upon receiving these calls, nurses typically visit a patient's room. These call records were categorized into two types: general and sensor calls. While the former is caused by a patient pressing a bed-attached button, the latter is triggered by sensors. We calculated the numbers of general and sensor calls per day for each shift.

We also consider weekends/holidays due to the reduction of admissions, surgeries, and tests. These were categorized as either weekday (0) or weekend/holiday (1).

### 2.3. Multiple Comparisons

To select the appropriate method for multiple comparisons, we checked the normality and homoscedasticity of nurses' walking distance for each shift (see Appendix C for details). Since the data did not exhibit normality and homoscedasticity, we used the Steel–Dwass–Critchlow–Fligner (SDCF) test [29], a nonparametric multiple comparison test robust against heteroscedasticity and not assuming normality.

#### 2.3.1. Effect Size and Power

In the SDCF test, we set the significance level at 0.05. Since a *p*-value, or statistical significance, does not measure the size of an effect or the importance of a result [30], we used Cliff's delta to quantify the effect size for group combinations with significant differences. Cliff's delta was chosen because our walking-distance data were neither normally distributed nor homoscedastic (Appendix C). As a distribution-free effect-size index based on stochastic dominance, Cliff's delta remains valid under such conditions, unlike parametric measures such as Cohen's *d*, which assume normality and equal variances. Moreover, Cliff's delta aligns naturally with the SDCF test, as both are rank-based statistics. Widely accepted benchmarks, that is, small (0.147), medium (0.33), and large (0.474), also allow for straightforward interpretation [31, 32].

In addition, we estimated the power of the test by simulation experiments, where the SDCF test was conducted 1000 times by generating random samples using the bootstrap method [33]. A nonparametric bootstrap approach was chosen because analytical power formulas are not available for the SDCF test under heteroscedastic, non-normal conditions. This method resamples from the observed data, preserving the empirical distribution, requires no distributional assumptions, and can be applied to any test statistic, including rank-based ones such as the SDCF statistic. For each group combination, we then calculated the proportion of tests where the *p*-value was less than the significance level.

#### 2.3.2. Programming

These tests were conducted using R 4.4.1. Specifically, the SDCF test was performed using the *pSDCFlig* function (method = “Asymptotic”) from the *NSM3* package, and Cliff's delta was computed using the *cliff.delta* function from the *effsize* package. Box plots were made using the *Seaborn* library in Python 3.12, where outliers are determined as points beyond the third quartile plus 1.5 times the interquartile range (IQR) or below the first quartile minus 1.5 times the IQR.

### 2.4. Regression Analysis

#### 2.4.1. Regression Model and Indicators for Evaluation

We used a multiple regression model to evaluate the impact of various factors on walking distance. We calculated the coefficients of the multiple regression model along with the 95% confidence interval and *p*-value. We also calculated the Root Mean Squared Error (RMSE) and *R*^2^ to evaluate the model's goodness of fit.

#### 2.4.2. Preprocessing

Due to system-related issues, we could not collect nurse call data on ward G, and thus, we removed data on ward G from the analysis. After that, we calculated the z-score for walking distance and removed data with z-scores greater than three. We applied the conventional threshold of |*z*| > 3, which corresponds to the outer 0.3% of a normal distribution and is commonly used, even for non-normal data, as a conservative criterion to detect extreme outliers. As a result, 5 of 888 day-shift observations (0.56%), 11 of 1002 long-day-shift observations (1.10%), and 11 of 1061 night-shift observations (1.04%) were removed. Thus, over 98% of the data in each dataset were retained, ensuring that extreme recording errors or device malfunctions did not excessively influence the regression analysis.

Next, we applied one-hot encoding to the categorical variables: clinical ladder level and ward. To avoid the dummy variable trap, one dummy variable was removed from each category, that is, ward D and Level I. We also standardized the continuous variables: age, years of service, the number of general/sensor calls, occupancy rate, bed utilization rate, the number of nurses, and PNR.

Finally, we calculated the variance inflation factor (VIF) to check for multicollinearity, and removed gender, years of service, occupancy rate, bed utilization rate, and the number of nurses (see Appendix D for details).

#### 2.4.3. Programming

The regression analysis was performed using Python 3.12. The VIF was calculated using the *variance_inflation_factor* function from the *statsmodels.stats.outliers_influence* module. The multiple regression analysis was conducted using the *OLS* function from the *statsmodels.api* module, setting the test size to 0.2.

### 2.5. Ethical Considerations

The study received approval from the Ethical Review Committee of the University of Osaka Hospital (No. 20444).

## 3. Results

### 3.1. Characteristics of Participants

We present the number of unique participants by nurses' personal information: gender, age, clinical ladder level, years of service, and ward, in Table 3.

### 3.2. Characteristics of Ward-Specific Data

**Figure 4:**
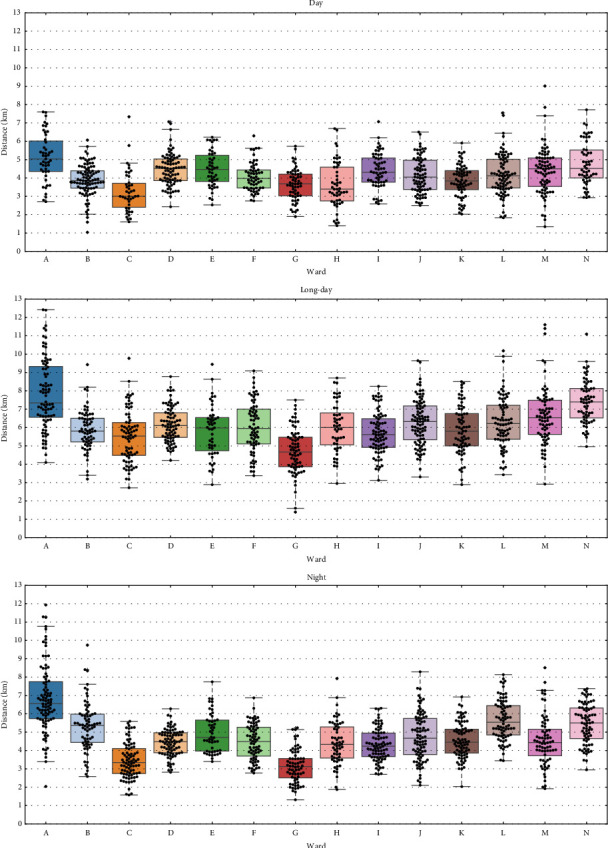
Nurses' walking distance by ward for each shift.

**Figure 5:**
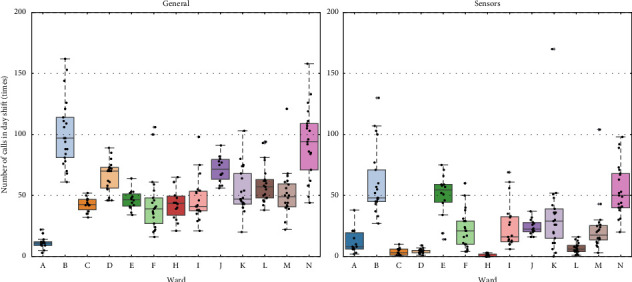
Daily total numbers of general and sensor calls during day shift by ward.

**Figure 6:**
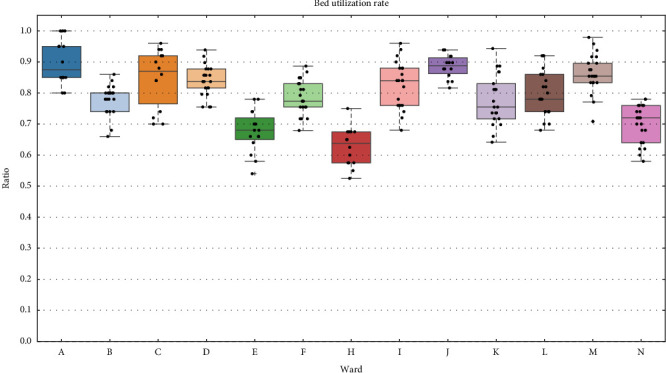
Bed utilization rate by ward.

We show box plots of ward-specific data in Figures 4, 5, 6.  Figure 4 shows nurses' walking distance by ward for the day, long-day, and night shifts. Ward A, an emergency ward, had the largest walking distance for all shifts, while ward C, a neurology ward, and ward G, a perinatal maternal and child medical center, had relatively smaller distances.  Figure 5 shows the daily total numbers of general and sensor calls during the day shift. Due to similar trends, figures for the long-day and night shifts are omitted. Wards B and N, both neurology-related wards, had the highest number of calls.  Figure 6 shows the bed utilization rates during each survey period, except for ward G. The bed utilization rates of wards A and J were high, while those of wards E, H, and N were low, relative to other wards.

### 3.3. Multiple Comparisons

**Figure 7:**
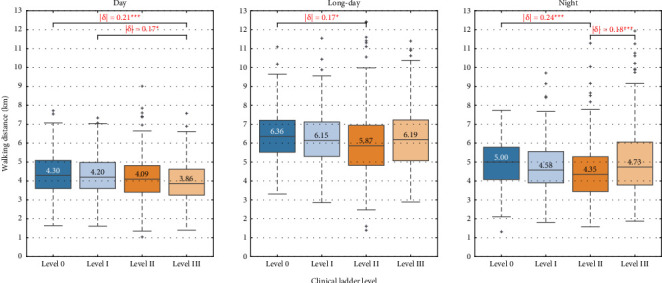
Multiple comparison among clinical ladder level groups.

**Figure 8:**
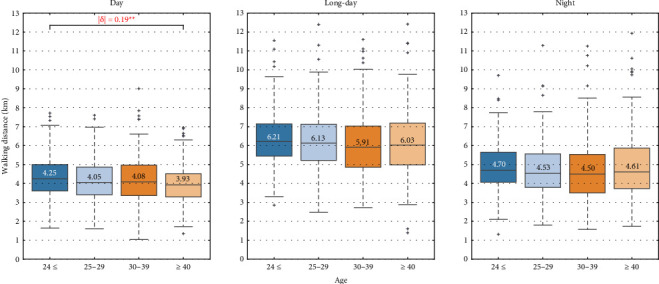
Multiple comparison among age groups.

**Figure 9:**
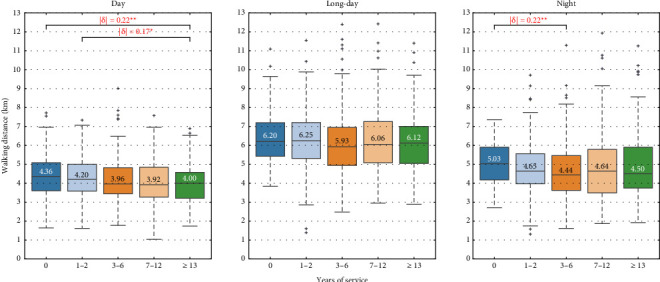
Multiple comparison among years of service groups.

We performed multiple comparisons among groups based on age, years of service, and clinical ladder level. Figures 7, 8, 9 show the box plots of walking distance for each shift by clinical ladder level, age, and years of service group, respectively. The median is depicted as text in the box, |δ| denotes the absolute value of Cliff's delta, and ∗, ∗∗, and ∗∗∗ represent significance levels of 0.05, 0.01, and 0.001, respectively.

In Figure 7, for the day shift, the walking distance for Level III is significantly less than that for Level 0 (|δ|=0.2, *p* < 0.001) and Level I (|δ|=0.17, *p*=0.02), with median differences of 0.44 and 0.34 kilometers. For the long-day shift, the walking distance for Level II is significantly less than for Level 0 (|δ|=0.17, *p*=0.015), with a median difference of 0.49 kilometers. For the night shift, the walking distance for Level II is significantly less than for Level 0 (|δ|=0.24, *p* < 0.001) and Level III (|δ|=0.18, *p* < 0.001), with median differences of 0.65 and 0.38 kilometers.

In Figure 8, for the day shift, the walking distance for those aged 40 and over is significantly less than for those aged 24 and under (|δ|=0.19, *p*=0.003), with a median difference of 0.32 kilometers. No significant differences in walking distance among age groups were found for the long-day and night shifts.

In Figure 9, for the day shift, the walking distance for those with 13 and over years of service is significantly less than for those with zero years (|δ| = 0.22, *p* = 0.005) and one to two years (|δ| = 0.17, *p* = 0.036), with median differences of 0.36 and 0.2 kilometers. No significant differences were found for the long-day shift. For the night shift, the walking distance for those with three to six years of service is significantly less than for those with zero years (|δ| = 0.22, *p* = 0.007), with a median difference of 0.59 kilometers.

Table 4 shows the summary of significant cases in the SDCF test for each shift, where the estimated power of the test calculated by simulation experiments is also shown.

In these cases, Cliff's delta ranges from 0.17 to 0.24, interpreted as a small to medium effect size. However, the estimated power is less than 0.7 for the following comparisons: Level 0 and Level II for the long-day shift, one to two years and 13 and over years of service for the day shift, and zero years and three to six years of service for the night shift, which indicates insufficient power to detect the differences between them.

### 3.4. Regression Analysis

We summarize the statistics of walking distance and the results of the multiple regression analysis for each shift in Tables 5 and 6.

For the day shift, coefficients for wards A, C, H, and general calls are significant, with their magnitudes more than 0.2 kilometers. Ward A and the number of general calls have a positive effect on walking distance, while wards C and H have a negative effect. Although the coefficient for Level III is significant, its magnitude is very small. The RMSE of the regression model is 0.97 kilometers, and the *R*^2^ is 0.07.

For the long-day shift, coefficients for wards A, B, and N, the number of general calls, and the weekend are significant, with their magnitudes more than 0.2 kilometers. Wards A and N and the number of general calls have a positive effect on walking distance, while ward B and the weekend have a negative effect. The RMSE of the regression model is 1.24 kilometers, and the *R*^2^ is 0.19.

For the night shift, coefficients for age, Level II, wards A, C, E, L, and N, the number of general calls, and PNR are significant, with their magnitudes more than 0.1 kilometers. Age, wards A, E, L, and N, the number of general calls, and PNR have a positive effect on walking distance, while Level II and ward C have a negative effect. The RMSE of the regression model is 1.10 kilometers, and the *R*^2^ is 0.30.

## 4. Discussion

### 4.1. Validity of Walking Distance for Each Shift

In this study, we used the preinstalled “Health” application on iOS devices unlike previous studies that used pedometers [23], RFID tags [19], and smart bands [22]. Although participant-specific stride lengths or heights were not entered into the devices, Apple's Core Motion algorithm estimates walking distance based on internally calibrated stride models and step counts. Recent validation studies have shown that iPhones can estimate step counts with high accuracy under free-living conditions, reporting a mean absolute percentage error below 4% when compared with direct observation [34]. However, the default distance estimation is known to overestimate walking distance by approximately 43% due to generalized stride-length assumptions [35]. Despite this limitation, our primary focus was on relative differences in walking distance across shift types rather than on absolute values. Therefore, any systematic overestimation would have occurred uniformly across groups and would not have biased between-group comparisons.

As shown in Table 5, the mean ± standard deviation of walking distance for 8-h day shifts, 12-h day shifts, and 12-h night shifts were 4.17 ± 1.11, 6.18 ± 1.55, and 4.76 ± 1.48 kilometers, respectively, with medians of 4.13, 6.09, and 4.61 kilometers. When compared to Welton et al., who reported 6.75 ± 2.25 kilometers for 12-h day shifts and 6.36 ± 2.25 kilometers for 12-h night shifts [23], our results for the 12-h day shift are comparable, although slightly shorter for the night shift. When compared to Hendrich et al., who reported 4.82 and 3.54 kilometers per 10 hours for day and night shifts [19], our results are relatively consistent. On the other hand, compared to Chang and Cho, who reported 5.97 ± 2.32 kilometers for 8-h day shifts and 5.32 ± 2.51 kilometers for 9-h night shifts [22], our results show shorter walking distance for both day and night shifts. This discrepancy may be due to their study being limited to a younger demographic, given the fact that younger nurses tend to have longer walking distance, as discussed in the next section.

Furthermore, the results of this study indicate that walking distance was longest for the long-day shift, followed by the night shift, and then the day shift. It is reasonable that the long-day shifts, which have longer working hours and include daytime activities involving various events, result in the longest walking distance. Additionally, the order of the day and night shifts is also reasonable because, although workloads decrease during night shifts, the number of nurses is reduced accordingly, and the working hours are longer.

### 4.2. Multiple Comparisons

#### 4.2.1. Clinical Ladder Levels

For the day shift, the walking distance of the Level III group was significantly shorter than that of the Level 0 and Level I groups. Level II and III nurses, who have acquired higher nursing skills, are more likely to be in charge of critically ill patients in rooms closer to the nurse station. Conversely, Level 0 and Level I nurses often care for patients with milder conditions who are in rooms located further from the nurse station, and they also engage in tasks such as surgery preparation and escorting patients for tests. Therefore, task variations associated with clinical ladder levels can affect walking distance.

For the long-day shift, unlike the day shift, significant differences were not found between the Level III and Level 0 groups, and between the Level III and Level I groups. This may be due to an increase in the number of assigned patients per nurse (PNR) as a result of the reduction in nurse numbers after 5:00 p.m. This PNR increase might cause Level III nurses to assist Level 0 or Level I nurses with uncompleted tasks, such as checking temperature, administering medication, and monitoring food intake, thus increasing the walking distance of the Level III group.

For the night shift, the walking distance of the Level II group was significantly shorter than that of both the Level 0 and Level III groups. Note here that in many Japanese hospitals, including our target hospital, to ensure safety, ward managers create shift schedules so that the nurses' clinical ladder levels are as evenly distributed as possible when assigning long-day and night shifts; for example, four nurses are assigned, one from each level. Given this practice, and considering that the number of nurses for night shifts is fewer than that for daytime shifts, it increases the likelihood of Level III nurses assisting Level 0 or Level I nurses, thereby increasing their walking distance. The significant difference between the Level 0 and Level II groups may be attributed not only to task variations based on clinical ladder levels, but also to redundant movements among the Level 0 group due to their lack of experience in night shifts, such as repeatedly returning to the nurse station to retrieve forgotten items.

Next, we focus on the difference between the Level 0 and Level I groups. For the day shift, the walking distance of both the Level 0 and Level I groups was significantly longer than that of the high-skilled (Level III) group, whereas for the night shift, only the Level 0 group showed significantly longer distances than the high-skilled (Level II) group. Given that, as shown in Figure 3, most of the nurses with zero years and one to two years of service belong to the Level 0 and Level I groups, respectively, after zero year of service, when rookie nurses (Level 0 and 0 years of service) advance to Level I, the significant difference in walking distance compared to the high-skilled group for the night shift is no longer observed. This finding suggests that rookie nurses might adapt to the night shift faster than to the day shift. This is because the number of tasks, such as following doctors' orders, distributing oral medication, sending and receiving patients for tests and surgeries, preparing infusions, and responding to nurse calls, generally decreases during the night shift, making the tasks relatively simpler compared to the day shift. Recall here that the estimated power of the significant difference between the Level 0 and Level I groups for the day shift is low, at 0.625, and thus, this suggestion should be treated with caution.

#### 4.2.2. Age

For the day shift, the walking distance of nurses aged 40 and over was significantly shorter than that of those aged 24 and under. As shown in Figure 3, nurses aged 24 and under largely belong to the Level 0 group, while those aged 40 and over belong to the Level III group. Therefore, the observed significant difference can be explained by both task variations associated with clinical ladder levels and younger nurses' lack of experience in night shifts, as mentioned above.

#### 4.2.3. Years of Service

For the day shift, the walking distance of nurses with 13 and over years of service was significantly shorter than that of those with zero years and one to two years of service. Given that, as shown in Figure 3, most of the nurses with 13 and over years of service belong to the Level III group, these significant differences can also be explained by the abovementioned task variations associated with clinical ladder levels.

### 4.3. Regression Analysis

The intercepts for the day, long-day, and night shifts were 4.45, 6.06, and 4.4 kilometers, respectively, aligning closely with the means shown in Table 5. Therefore, we evaluate the impact of each factor on walking distance by examining the regression coefficients.

#### 4.3.1. Age and Clinical Ladder Levels

For the day shift, the coefficient for Level III was significant, and for the night shift, the coefficients for age and Level II were significant. These reasons are the same as those given in the previous subsection. However, their magnitudes were small, that is, less than 0.25 kilometers. Despite the statistical significance with small to medium effect sizes found by the SDCF test among groups based on clinical ladder levels and age, these factors had minimal impact on walking distance compared to others.

#### 4.3.2. Wards

For the day shift, coefficients for wards A, C, and H were significant, and for the long-day shift, those for wards A, B, and N were significant, with all magnitudes for both shifts exceeding 0.8 kilometers. For the night shift, coefficients for wards A, C, E, L, and N were significant, with magnitudes exceeding 0.5 kilometers. Only ward A had a significant impact on walking distance across all shifts, with large positive magnitudes. In Figure 4, ward A, an emergency ward with an outpatient clinic, had clearly larger walking distance than other wards. This can be due to the survey period coinciding with the highest ambulance admissions and the unique layout for emergency care.

We focus on wards B and N, which are neurology-related wards. Although there is a difference between medical and surgical wards, their nurse call frequency is very high as shown in Figure 5. However, for the long-day shift, ward B has a significant negative effect, whereas ward N has a positive effect, with magnitudes greater than 0.8. Given that only surgical ward N has a significant positive effect on walking distance for the night shift, the difference in impacts between wards B and N may stem from the difference in the number of surgeries. If a surgery is completed after 5:00 p.m., nurses on the long-day or night shift must pick up patients from the operating room. In surgical wards, this sometimes happens, possibly leading to an increase in walking distance.

Next, we focus on wards K and L, which are both gastrointestinal surgery wards. For the night shift, only ward L has a significant positive effect, with a large magnitude of 1.22 km. Since the number of nurse calls, bed utilization rates, the number of nurses on night shift, and other environmental factors are almost the same, the difference can likely be attributed to patient severity or some operational differences, though the specific factors remain unclear.

Lastly, we focus on wards C, E, and H. For both day and night shifts, ward C has a significant negative effect, with medium to large magnitudes of 1.08 and 0.58 kilometers, respectively, despite the high bed utilization rate, as shown in Figure 6. Considering that, as described in Section 2.2, ward C has six beds for the high care unit (3:1), and these rooms are arranged adjacently (corresponding to beacons 13–17 and 27 in Figure 2), this may be due to the shorter walking distance for nurses assigned to the high care unit, as they spend a substantial amount of time in these rooms. For the night shift only, ward E has a significant positive effect, with a medium magnitude of 0.59 kilometers. This is likely because the number of intravenous infusions was extremely high, given the nature of the patients' illnesses, which had a notable impact during the night shift with fewer nurses. For the day shift, ward H has a significant negative effect, with a large magnitude of 0.83 kilometers. As shown in Figures 4 and 5, the relatively small numbers of general and sensor calls, along with the low bed utilization rate, likely contributed to the shorter walking distance.

In summary, these significant coefficients for various wards indicate that the ward characteristics substantially affect walking distance, which is consistent with the previous study [22].

#### 4.3.3. Number of Nurse Calls

The number of general calls had a significant positive impact on walking distance across all shifts, with their magnitudes greater than those for clinical ladder levels. In contrast, sensor calls did not show significant effects in any shift. As shown in Figure 5, general calls are more frequent than sensor calls, with a broader IQR, which can explain why general calls more substantially affect walking distance than sensor calls. The small impact of sensor calls might seem incongruent considering the principle mentioned in Section 2.2, which states that nurses are generally required to visit a patient's room upon receiving general or sensor calls. A practical interpretation of this incongruity is that sensor calls often occur in quick succession and are handled together in a single visit, thereby reducing their overall impact.

Studies on nurse call analysis indicate periodicity in call frequency [36] and patient-specific variations [37], suggesting that patients with high call frequencies on one day tend to have high call frequencies on other days as well. These insights, combined with the above result, suggest that evenly distributing patients with high call frequencies could help balance nurses' workloads and reduce walking distance.

#### 4.3.4. PNR

The PNR had a significant positive impact on walking distance only for the night shift. In Figure 6, most of the wards have an IQR range exceeding 0.1, which means that bed utilization rates during the experiments varied widely; for example, in ward C, there are days when it exceeds 0.9, while on other days it falls below 0.75. The PNR is very sensitive to fluctuations in bed utilization rates due to the small number of nurses on night shifts. For example, in ward I, a 0.1 increase in the bed utilization rate is equivalent to an additional 1.25 patients per nurse, assuming 50 beds and four nurses. Therefore, an increase in PNR leads to an increase in workloads per nurse, such as the number of nurse calls they need to respond to, which can increase walking distance. This is consistent with Welton's results [23].

#### 4.3.5. Weekends/Holidays

Weekends had a significant negative impact on walking distance only for the long-day shift, reflecting the reduced hospital operations such as admissions, surgeries, and tests on weekends. Since the number of day-shift nurses on weekends is reduced compared to weekdays according to those reduced operations, resulting in the workload per nurse remaining approximately the same, the coefficient may not be significant for the day shift. For the night shift, the tasks do not change largely compared to weekdays, thus not affecting walking distance significantly.

#### 4.3.6. Model Evaluation

Despite incorporating more variables than the previous study [22], both RMSE and *R*^2^ values remain low across all shifts. This suggests that these variables alone are insufficient to fully explain the complexities of nursing tasks. The result that *R*^2^ values are higher in the order of the day, long-day, and night shifts further supports this interpretation. To improve the accuracy of the regression models for estimating walking distance, it is necessary to consider additional factors such as patient severity, care levels, the number of infusions including blood transfusions, and the frequency of blood glucose measurements. Moreover, accounting for random effects specific to individual nurses could enhance the model's accuracy [38, 39].

### 4.4. Strategies for Reducing Physical Burdens on Nurses

This study suggests that Level III nurses often assist Level 0 or Level I nurses, particularly during night shifts, thereby potentially increasing their walking distance. As shown in Figure 3, over half of Level III nurses are aged 40 and above, yet there are no significant differences in walking distance between those aged 40 and above and those aged 24 and under. This indicates that Level III nurses provide assistance regardless of age. Although chronological age itself is not a strong predictor of walking distance, Level III nurses—who are often older and more experienced—tend to take on supporting roles that may increase their physical burden. Given the expected acceleration of nursing workforce aging in many developed countries, addressing these role-based burdens is essential for improving the long-term sustainability of night shift staffing.

To reduce the physical strain on older nurses, it may be helpful to reconsider the approach to task and patient assignment. For example, in cases such as our target wards, where a primary nursing system is employed, it is conceivable that assigning specific tasks such as vital sign measurement, cleansing, and medication administration to a few nurses could simplify the complex tasks of the other nurses who perform primary nursing. This hybrid strategy of primary nursing and function-based nursing could reduce the likelihood of high-skilled nurses assisting rookie nurses and may also reduce their walking distance.

The period when rookie nurses begin night shifts varies by hospitals and wards. At the target hospital, rookies typically start night shifts three to six months after employment. During the first several months of the night shift, rookies are allocated fewer night shifts than other nurses, that is, a few times per month. If the period when rookies work fewer night shifts is prolonged due to ward customs or ward managers' attitudes toward safety, nursing quality, and competency assessment, it not only delays rookies' adaptation to night shifts but also increases the night shift burden on other nurses, particularly older ones, potentially leading to their early resignations or withdrawals from night shifts. Therefore, considering our suggestion that rookies can adapt faster to night shifts than day shifts, it may be beneficial to reconsider the practice of delaying night shift allocations for rookies until they have mastered day-shift tasks. If this practice is changed, however, it is crucial for ward managers to adjust patient assignments according to the developmental stages of rookies' nursing skills to ensure safety and quality of nursing care and prevent medical accidents. It is also important to carefully monitor the physical and mental health of rookies to prevent them from being overwhelmed by the pressures of nursing care and the expectations of senior staff for rapidly improving their nursing skills.

From a rookie education perspective, two competency-based strategies may help facilitate safer and more effective adaptation to night shifts. First, early shadow night shifts can be introduced within the first two months of employment. By pairing each rookie with an experienced preceptor during night shifts, they can observe and assist with night-shift workflows such as reduced staffing handovers, vital-sign rounds, and nurse calls, without being primarily responsible for patient care. This early, low-stakes exposure helps familiarize them with the circadian and cognitive demands of night work while providing psychological safety and real-time feedback. Second, the strategic use of late (evening) shifts, typically from 1:00 p.m. to 10:00 p.m., can serve as an intermediate step before full night shifts. The evening period often involves a temporary increase in patient assignments, offering opportunities for rookies to practice managing heavier workloads, prioritizing tasks, and documenting under time pressure. These experiences enhance their readiness for night shifts by improving both their workload tolerance and their physiological adjustment to later hours. Implementing these structured supports may accelerate adaptation, reduce the burden on senior staff, and improve long-term nurse retention and shift flexibility.

From the regression analysis, we found that ward characteristics have a greater impact on walking distance than nurse-specific characteristics. Moreover, the comparison in Figure 4 reveals that nurses' walking distance vary notably among wards. These findings suggest that ward managers should develop strategies to reduce physical burdens on nurses through the following two steps. In step 1, to reduce absolute walking distance in each ward, ward managers should analyze ward-specific data from multiple perspectives, such as the number of nurse calls, bed utilization rates, the number of surgeries and tests, the number of infusions, the severity of illness, the level of care, and the frequency of blood glucose measurements, and understand the characteristics of each ward in detail by comparing them with other wards. Based on the analysis results, ward managers should reconsider nursing care systems [40], that is, the primary nursing system, the team nursing system, and the modular (cell) nursing system, in accordance with the characteristics of each ward. For example, in high call frequency wards, ward managers should consider introducing a nursing care system that can reduce nurse call frequency, such as the cell nursing system [41]. In step 2, as discussed above, ward managers should reconsider the allocation of tasks and patients according to the clinical ladder levels in order to balance workloads on nurses within their own ward. Note that the order of these steps is very important to perform this strategy effectively.

In addition, when discussing the reshuffling of older or physically weaker nurses, human resource managers should consider the high physical demands associated with ward characteristics, such as high call frequency and emergency wards.

### 4.5. Limitations

In this study, walking distance data were collected by a preinstalled application on iOS devices. Although the results of mean and median walking distance were relatively consistent with other studies, there may be measurement errors. Almost all eligible staff nurses in the target ward took part in the study, suggesting that selection bias within the hospital is unlikely. However, the study was conducted at a single Japanese national university hospital, where staffing ratios, patient acuity, and ward layout may differ from those in community or private hospitals. This single-site design limits the generalizability of the results. Multisite studies are needed to verify whether the shift-specific patterns observed here apply in other healthcare settings.

## 5. Conclusion

This study used nurses' walking distance data from the 14-ward survey in a large acute care hospital using an unmanned time study method with beacons and mobile devices. To identify factors affecting nurses' walking distance, we conducted multiple comparisons using the SDCF test and multiple regression analysis. Our results of the statistical analyses revealed that clinical ladder levels significantly impact walking distance, although the magnitude is smaller compared to other factors. The results also indicated that ward characteristics substantially affected walking distance and that the number of general calls, weekends, and PNR were significant factors affecting walking distance. These findings suggest that ward managers should thoroughly understand their ward's characteristics by comparing various data with other wards and reconsider approaches to task assistance for less experienced nurses and night shift allocations for rookie nurses to effectively reduce physical burdens on nurses, especially older ones. These results contribute to the understanding of factors affecting nurses' walking distance and support the development of evidence-based strategies for improving work environments in acute care settings.

Future research should examine the efficiency of nurses' movement lines among clinical ladder levels by analyzing data on beacon's RSSIs. This would provide a more comprehensive understanding of not only the walking distance, but also the effectiveness of nurses' workflow. In particular, identifying redundant or inefficient movements may offer insights into how certain tasks or workflows contribute to increased physical burden. This line of inquiry may help support novice nurses in managing tasks more effectively and facilitate task automation through ICT-based innovations.

## Figures and Tables

**Table 1:** Overview of the 14-ward survey.

Ward	Department	Period	Days	Beds
A	Emergency medicine	January 13–26, 2023	14	16 (4:1), 4 (2:1)
B	Neurology/geriatric and hypertension medicine	November 7–27, 2023	21	50 (7:1)
C	Cardiology	August 1–14, 2022	14	44 (7:1), 6 (3:1)
D	Hematology oncology	August 1–21, 2023	21	49 (7:1)
E	Gastroenterology	July 1–15, 2023	15	50 (7:1)
F	Diabetes, endocrinology, metabolism/immunology	September 21, 2023–October 11, 2023	21	53 (7:1)
G	Perinatal maternal and child medical center	July 3–16, 2023	14	20 (7:1), 3 (3:1)
H	Pediatric surgery	Jun 9–22, 2022	14	40 (7:1)
I	Breast, endocrine surgery/plastic surgery/dermatology	November 1–21, 2022	21	50 (7:1)
J	Cardiovascular surgery	March 15–28, 2023	14	43 (7:1), 6 (3:1)
K	Gastrointestinal surgery	February 26, 2024–March 17, 2024	21	53 (7:1)
L	Gastrointestinal surgery	January 12, 2024–February 1, 2024	21	50 (7:1)
M	Urology	February 6–26, 2023	21	48 (7:1)
N	Neurosurgery	September 9–29, 2022	21	50 (7:1)

*Note:* In the “beds” column, the number represents the number of beds, and the ratios in parentheses indicate the number of patients per nurse. Wards C, G, and J have beds for high care units (3:1) in addition to general beds (7:1).

**Table 2:** Key criteria for each clinical ladder level.

Level	Description
0	Novice in training in basic nursing practice
I	Advanced beginner can demonstrate marginally acceptable performance in basic nursing practice
II	Competent has been on the job two to 3 years and can capture their practice in terms of long-term plans and goals
III	Proficient/expert can empirically assess the overall situation

**Table 3:** Number of unique participants by nurses' personal information.

Personal information	Group	Day: 343		Long-day: 393		Night: 393	
		*N*	%	*N*	%	*N*	%
Gender	Female	321	93.6	363	92.4	363	92.4
	Male	22	6.4	30	7.6	30	7.6

Age	24 ≦	88	25.7	95	27.7	94	27.4
	25–29	109	31.8	124	36.2	123	35.9
	30–39	82	23.9	94	27.4	95	27.7
	≧ 40	64	18.7	80	23.3	81	23.6

Clinical ladder level	Level 0	66	19.2	67	19.5	66	19.2
	Level I	78	22.7	88	25.7	88	25.7
	Level II	100	29.2	121	35.3	121	35.3
	Level III	99	28.9	117	34.1	118	34.4

Years of service	0	47	13.7	48	14.0	47	13.7
	1–2	94	27.4	105	30.6	105	30.6
	3–6	92	26.8	105	30.6	104	30.3
	7–12	53	15.5	68	19.8	69	20.1
	≧ 13	57	16.6	67	19.5	68	19.8

Ward	A	30	8.7	41	11.9	40	11.6
	B	26	7.5	25	7.2	26	7.5
	C	25	7.2	36	10.4	36	10.4
	D	26	7.5	27	7.8	27	7.8
	E	17	4.9	23	6.7	21	6.1
	F	23	6.7	24	7.0	25	7.2
	G	17	4.9	29	8.4	29	8.4
	H	23	6.7	25	7.2	26	7.5
	I	24	7.0	25	7.2	25	7.2
	J	37	10.7	40	11.6	39	11.3
	K	23	6.7	23	6.7	24	7.0
	L	26	7.5	25	7.2	25	7.2
	M	27	7.8	26	7.5	26	7.5
	N	21	6.1	24	7.0	25	7.2

**Table 4:** Summary of significant cases in the SDCF test.

Group	Shift	Category 1	Category 2	*p*-value	Effect size	Power
Clinical ladder	Day	Level 0	Level III	< 0.001	0.2	0.909
	Day	Level I	Level III	0.02	0.17	0.625
	Long-day	Level 0	Level II	0.015	0.17	0.67
	Night	Level 0	Level II	0.001	0.24	0.973
	Night	Level II	Level III	< 0.001	0.18	0.923

Age	Day	24 ≦	≧ 40	0.003	0.19	0.838

Years of service	Day	0	≧ 13	0.005	0.22	0.777
	Day	1-2	≧ 13	0.036	0.17	0.562
	Night	0	3–6	0.007	0.22	0.754

*Note:* Effect size (the absolute value of Cliff's delta) and power (the estimated power of the SDCF test).

**Table 5:** Statistics of walking distance (km).

Shift	*N*	Mean	SD	Min	1Q	Median	3Q	Max
Day	883	4.17	1.11	1.04	3.43	4.13	4.86	9.01
Long-day	991	6.18	1.55	1.39	5.12	6.09	7.11	12.42
Night	1050	4.76	1.48	1.31	3.78	4.61	5.62	11.93

*Note:* 1Q and 3Q (the first and third quartiles).

Abbreviation: SD = standard deviation.

**Table 6:** Results of the multiple regression analysis.

Variables	Day			Long-day			Night		
	Coef	*p*-value	95% CI	Coef	*p*-value	95% CI	Coef	*p*-value	95% CI
Intercept	4.45	0	(3.81, 5.09)	6.06	0	(5.57, 6.55)	4.4	0	(3.91, 4.88)
Age	0	0.952	(−0.12, 0.11)	−0.1	0.194	(−0.24, 0.05)	**0.14**	**0.017**	**(0.03, 0.26)**
Level 0	−0.23	0.858	(−0.49, 0.02)	−0.11	0.094	(−0.39, 0.17)	−0.24	0.076	(−0.48, 0)
Level II	−0.4	0.076	(−0.7, −0.1)	0.12	0.443	(−0.25, 0.48)	**−0.23**	**0.046**	**(**−**0.52, 0.07)**
Level III	**0.02**	**0.01**	**(**−**0.23, 0.27)**	0.27	0.524	(−0.05, 0.59)	0.23	0.133	(−0.03, 0.49)
General calls	**0.28**	**0.001**	**(0.11, 0.44)**	**0.25**	**0.001**	**(0.1, 0.39)**	**0.18**	**0.015**	**(0.04, 0.32)**
Sensor calls	−0.11	0.091	(−0.24, 0.02)	−0.05	0.467	(−0.17, 0.08)	0.04	0.563	(−0.1, 0.19)
PNR	−0.05	0.868	(−0.58, 0.49)	0.13	0.424	(−0.18, 0.43)	**0.36**	**0.017**	**(0.07, 0.66)**
Weekend	−0.11	0.484	(−0.44, 0.21)	**−0.36**	**0.001**	**(**−**0.57,** −**0.14)**	0.01	0.919	(−0.16, 0.18)
Ward A	**1.24**	**0.001**	**(0.52, 1.97)**	**1.8**	**< 0.001**	**(0.94, 2.66)**	**2.85**	**< 0.001**	**(2.04, 3.67)**
Ward B	−0.44	0.055	(−0.88, 0.01)	**−0.81**	**0.013**	**(**−**1.45,** −**0.17)**	0.3	0.339	(−0.32, 0.91)
Ward C	**−1.08**	**< 0.001**	**(**−**1.55,** −**0.61)**	−0.52	0.061	(−1.06, 0.02)	**−0.58**	**0.017**	**(**−**1.06,** −**0.1)**
Ward E	0.32	0.205	(−0.18, 0.81)	−0.05	0.87	(−0.67, 0.56)	**0.59**	**0.021**	**(0.09, 1.09)**
Ward F	−0.11	0.615	(−0.53, 0.31)	−0.18	0.485	(−0.69, 0.33)	−0.13	0.544	(−0.55, 0.29)
Ward H	**−0.83**	**0.002**	**(**−**1.35,** −**0.31)**	−0.24	0.475	(−0.9, 0.42)	0.47	0.116	(−0.12, 1.05)
Ward I	0.16	0.439	(−0.25, 0.58)	−0.42	0.091	(−0.9, 0.07)	−0.27	0.186	(−0.67, 0.13)
Ward J	−0.23	0.322	(−0.69, 0.23)	0.08	0.786	(−0.5, 0.67)	0.5	0.081	(−0.06, 1.05)
Ward K	−0.41	0.086	(−0.88, 0.06)	−0.36	0.192	(−0.89, 0.18)	−0.11	0.616	(−0.52, 0.31)
Ward L	−0.12	0.518	(−0.48, 0.24)	−0.04	0.865	(−0.51, 0.43)	**1.22**	**< 0.001**	**(0.82, 1.62)**
Ward M	0	0.989	(−0.38, 0.38)	0.33	0.176	(−0.15, 0.81)	0.08	0.721	(−0.34, 0.49)
Ward N	0.42	0.094	(−0.07, 0.9)	**0.94**	**0.002**	**(0.34, 1.53)**	**0.84**	**0.003**	**(0.29, 1.38)**

*Note:* Coef (coefficient of the multiple regression model), bold values (significant at the 0.05 level).

## Data Availability

The access data used to support the findings of this study have not been made available to protect personal information.

## Supporting Information

This paper includes Appendices A, B, C, and D in Supporting Information. Appendix A presents the sample size for each ward and group, confirming limited influence from any single ward. Appendix B shows the distribution of records per nurse, indicating balanced data contributions. Appendix C examines normality and homoscedasticity, supporting the use of nonparametric methods. Appendix D reports variance inflation factors used to assess multicollinearity in regression analysis.

## References

[1] https://www.mhlw.go.jp/content/10800000/001118192.pdf.

[2] Trinkoff A. M., Storr C., Lipscomb J. A. (2001). Physically Demanding Work and Inadequate Sleep, Pain Medication Use, and Absenteeism in Registered Nurses. *Journal of Occupational and Environmental Medicine*.

[3] Trinkoff A. M., Lipscomb J. A., Geiger-Brown J., Storr C. L., Brady B. A. (2003). Perceived Physical Demands and Reported Musculoskeletal Problems in Registered Nurses. *American Journal of Preventive Medicine*.

[4] Trinkoff A. M., Le R., Geiger-Brown J., Lipscomb J., Lang G. (2006). Longitudinal Relationship of Work Hours, Mandatory Overtime, and On-Call to Musculoskeletal Problems in Nurses. *American Journal of Industrial Medicine*.

[5] Winwood P. C., Lushington K. (2006). Disentangling the Effects of Psychological and Physical Work Demands on Sleep, Recovery and Maladaptive Chronic Stress Outcomes Within a Large Sample of Australian Nurses. *Journal of Advanced Nursing*.

[6] Hayes L. J., O’Brien-Pallas L., Duffield C. (2012). Nurse Turnover: A Literature Review-An Update. *International Journal of Nursing Studies*.

[7] Hayward D., Bungay V., Wolff A. C., MacDonald V. (2016). A Qualitative Study of Experienced Nurses’ Voluntary Turnover: Learning From Their Perspectives. *Journal of Clinical Nursing*.

[8] Lu H., Barriball K. L., Zhang X., While A. E. (2012). Job Satisfaction Among Hospital Nurses Revisited: A Systematic Review. *International Journal of Nursing Studies*.

[9] Ki J., Ryu J., Baek J., Huh I., Choi-Kwon S. (2020). Association Between Health Problems and Turnover Intention in Shift Work Nurses: Health Problem Clustering. *International Journal of Environmental Research and Public Health*.

[10] Cho S. H., Park M., Jeon S. H., Chang H. E., Hong H. J. (2014). Average Hospital Length of Stay, Nurses’ Work Demands, and Their Health and Job Outcomes. *Journal of Nursing Scholarship*.

[11] Letvak S. (2013). We Cannot Ignore Nurses’ Health Anymore: A Synthesis of the Literature on Evidence-based Strategies to Improve Nurse Health. *Nursing Administration Quarterly*.

[12] Mc Carthy V. J. C., Wills T., Crowley S. (2018). Nurses, Age, Job Demands and Physical Activity at Work and at Leisure: A Cross-Sectional Study. *Applied Nursing Research*.

[13] Harrison L., Nixon G. (2002). Nursing Activity in General Intensive Care. *Journal of Clinical Nursing*.

[14] Ampt A., Westbrook J., Creswick N., Mallock N. (2007). A Comparison of Self-Reported and Observational Work Sampling Techniques for Measuring Time in Nursing Tasks. *Journal of Health Services Research & Policy*.

[15] Abbey M., Chaboyer W., Mitchell M. J. C. (2012). Understanding the Work of Intensive Care Nurses: A Time and Motion Study. *Australian Critical Care*.

[16] Sinsky C., Colligan L., Li L. (2016). Allocation of Physician Time in Ambulatory Practice: A Time and Motion Study in 4 Specialties. *Annals of Internal Medicine*.

[17] Yu P., Song L., Qian S. (2019). Work Pattern of Neurology Nurses in a Chinese Hospital: A Time and Motion Study. *Journal of Nursing Management*.

[18] Ehrler F., Wu D. T. Y., Ducloux P., Blondon K. (2021). A Mobile Application to Support Bedside Nurse Documentation and Care: A Time and Motion Study. *JAMIA Open*.

[19] Hendrich A., Chow M. P., Skierczynski B. A., Lu Z. (2008). A 36-Hospital Time and Motion Study: How Do Medical-Surgical Nurses Spend Their Time?. *The Permanente Journal*.

[20] Morita T., Taki K., Fujimoto M., Suwa H., Arakawa Y., Yasumoto K. (2018). Beacon-Based Time-Spatial Recognition Toward Automatic Daily Care Reporting for Nursing Homes. *Journal of Sensors*.

[21] Nakashima K., Inoue Y., Fukushige H., Ishii A. (2024). An Estimation and Visualization Method for the Movement Lines of Nurses in an Unattended Time and Motion Study with Beacons and Mobile Devices. *Journal of Nursing Science and Engineering*.

[22] Chang H. E., Cho S. H. (2022). Nurses’ Steps, Distance Traveled, and Perceived Physical Demands in a Three-Shift Schedule. *Human Resources for Health*.

[23] Welton J. M., Decker M., Adam J., Zone-Smith L. (2006). How Far Do Nurses Walk?. *Medsurg Nursing: Official Journal of the Academy of Medical-Surgical Nurses*.

[24] Yi L., Seo H. B. (2012). The Effect of Hospital Unit Layout on Nurse Walking Behavior. *HERD: Health Environments Research & Design Journal*.

[25] Copeland D., Chambers M. (2017). Effects of Unit Design on Acute Care Nurses’ Walking Distances, Energy Expenditure, and Job Satisfaction: A Pre-Post Relocation Study. *HERD: Health Environments Research & Design Journal*.

[26] Shakil M. (2022). Comparative Analysis of Inpatient Ward Typologies for Space Program and Nurses’ Walking Distance. *Journal of Development and Social Sciences*.

[27] Health. https://www.apple.com/jp/ios/health/.

[28] Benner P. (1982). From Novice to Expert. *AJN, American Journal of Nursing*.

[29] Douglas C. E., Michael F. A. (1991). On Distribution-Free Multiple Comparisons in the One-Way Analysis of Variance. *Communications in Statistics-Theory and Methods*.

[30] Wasserstein R. L., Lazar N. A. (2016). The ASA Statement on *p*-Values: Context, Process, and Purpose. *The American Statistician*.

[31] Macbeth G., Razumiejczyk E., Ledesma R. (2010). Cliff’s Delta Calculator: A Non-Parametric Effect Size Program for Two Groups of Observations. *Universitas Psychologica*.

[32] Meissel K., Yao E. S. (2024). Using Cliff’s Delta as a Non-Parametric Effect Size Measure: an Accessible Web App and R Tutorial. *Practical Assessment, Research and Evaluation*.

[33] Chernick M. R. (2012). Resampling Methods. *WIREs Data Mining and Knowledge Discovery*.

[34] Judice P., Fouto J., Horta M., Pereira S. (2024). Validity of iPhone Health Application Step Count in Semi Free-Living Conditions. *Acta IMEKO*.

[35] Ata R., Gandhi N., Rasmussen H. (2018). Clinical Validation of Smartphone-Based Activity Tracking in Peripheral Artery Disease Patients. *NPJ Digital Medicine*.

[36] Fukushige H., Ishii A., Inoue Y. (2021). Identifying Periodicity in Nurse Call Occurrence: Analysing Nurse Call Logs to Obtain Information for Data‐Based Nursing Management. *Journal of Nursing Management*.

[37] Noguchi H., Miyahara M., Kang S. I. Bayesian Statistic Model for Nurse Call Data Considering Time-Series, Individual Patient Variabilities and Massive Zero-Count Call Data.

[38] Yen S. C., Corkery M. B., Chui K. K., Manjourides J., Wang Y. C., Resnik L. J. (2015). Risk Adjustment for Lumbar Dysfunction: Comparison of Linear Mixed Models with and Without Inclusion of Between-Clinic Variation as a Random Effect. *Physical Therapy*.

[39] Swaminathan S. S., Berchuck S. I., Rao J. S., Medeiros F. A. (2024). Performance of Linear Mixed Models in Estimating Structural Rates of Glaucoma Progression Using Varied Random Effect Distributions. *Ophthalmology Science*.

[40] Prentice D., Moore J., Desai Y. (2021). Nursing Care Delivery Models and Outcomes: A Literature Review. *Nursing Forum*.

[41] Ikegawa M., Ohshima S., Suso K., Kurachi E. (2018). Case of Nursing Activity Improvement From Viewing of the Place of Work With Cell Nursing System. *Journal of Nursing Science and Engineering*.

