# Supplementary material for: Factors Affecting Nurses' Walking Distance: Age, Clinical Ladder Level, Wards, Nurse Calls, Weekend, and Patient-To-Nurse Ratio
Source: J Nurs Manag. 2025 Aug 28;2025:5540600. doi: 10.1155/jonm/5540600 (PMC12411061; doi:10.1155/jonm/5540600)
Supplement: Supporting Information — Additional supporting information can be found online in the Supporting Information section. [file 5540600.f1.docx]

#### **Supplementary Material**

#### **Sample Size for Each Ward and Group**

The sample size per group is shown in Table 7. For all groups, the sample size from each ward was less than $20$ percent of the total sample size for that group. This limitation on the influence of any specific ward ensures that the composition of subjects in each group can be considered nearly homogeneous.

Table 7: Sample Size for each ward and group

|  |  | Each ward’s ratio to the total sample size [$\boldsymbol{\%}$] | | | | | | | | | | | | | |
| --- | --- | --- | --- | --- | --- | --- | --- | --- | --- | --- | --- | --- | --- | --- | --- |
|  | **Total** | **A** | **B** | **C** | **D** | **E** | **F** | **G** | **H** | **I** | **J** | **K** | **L** | **M** | **N** |
| **Day** |  |  |  |  |  |  |  |  |  |  |  |  |  |  |  |
| **Total** | 883 | 5.3 | 10.3 | 4.8 | 9.2 | 5.2 | 6.6 | 6.6 | 5.8 | 6.5 | 8.5 | 7.2 | 9.3 | 8.8 | 6.0 |
| **Age** |  |  |  |  |  |  |  |  |  |  |  |  |  |  |  |
| 24 ≦ | 258 | 4.7 | 5.0 | 3.5 | 7.4 | 8.9 | 8.5 | 10.5 | 7.8 | 11.6 | 1.6 | 12.0 | 8.5 | 5.4 | 4.7 |
| 25 − 29 | 225 | 4.4 | 4.9 | 6.2 | 10.2 | 3.1 | 8.0 | 5.8 | 3.6 | 3.1 | 17.3 | 7.6 | 10.2 | 8.0 | 7.6 |
| 30 − 39 | 214 | 7.9 | 16.4 | 7.0 | 6.1 | 6.5 | 1.4 | 0.5 | 10.3 | 1.4 | 11.2 | 1.4 | 7.9 | 14.5 | 7.5 |
| ≧ 40 | 186 | 4.3 | 17.2 | 2.2 | 14.0 | 1.1 | 8.1 | 9.1 | 0.5 | 9.1 | 4.3 | 7.0 | 10.8 | 8.1 | 4.3 |
| **Clinical Ladder Level** | | |  |  |  |  |  |  |  |  |  |  |  |  |  |
| Level 0 | 214 | 5.1 | 4.7 | 3.3 | 8.4 | 9.3 | 6.1 | 9.3 | 9.3 | 12.6 | 3.7 | 10.3 | 6.1 | 7.9 | 3.7 |
| Level I | 153 | 3.9 | 5.9 | 6.5 | 9.2 | 4.6 | 10.5 | 11.1 | 3.3 | 2.6 | 7.8 | 9.8 | 11.8 | 8.5 | 4.6 |
| Level II | 261 | 4.2 | 15.3 | 5.0 | 12.3 | 3.4 | 4.2 | 5.4 | 1.9 | 5.0 | 16.1 | 1.9 | 4.6 | 13.8 | 6.9 |
| Level III | 255 | 7.5 | 12.5 | 4.7 | 6.7 | 3.9 | 7.1 | 2.7 | 8.2 | 5.1 | 5.1 | 8.6 | 15.3 | 4.7 | 7.8 |
| **Years of Service** | |  |  |  |  |  |  |  |  |  |  |  |  |  |  |
| 0 | 164 | 6.7 | 6.1 | 1.2 | 9.1 | 7.9 | 6.7 | 11.6 | 7.9 | 11.6 | 3.0 | 8.5 | 7.9 | 6.7 | 4.9 |
| 1 − 2 | 206 | 2.9 | 4.4 | 5.8 | 6.8 | 5.8 | 9.2 | 13.6 | 4.4 | 5.8 | 7.3 | 11.2 | 10.2 | 9.2 | 3.4 |
| 3 − 6 | 164 | 2.4 | 15.9 | 1.2 | 15.9 | 0.6 | 3.0 | 4.3 | 3.0 | 9.1 | 9.1 | 8.5 | 12.8 | 8.5 | 5.5 |
| 7 − 12 | 211 | 5.2 | 11.8 | 6.6 | 7.1 | 3.3 | 6.6 | 1.4 | 3.3 | 3.8 | 17.1 | 5.7 | 6.6 | 11.8 | 9.5 |
| ≧ 13 | 138 | 10.9 | 15.2 | 8.7 | 8.0 | 9.4 | 6.5 | 0.7 | 12.3 | 2.2 | 2.9 | 0.7 | 9.4 | 6.5 | 6.5 |
| **Long−Day** | |  |  |  |  |  |  |  |  |  |  |  |  |  |  |
| **Total** | 991 | 8.5 | 6.7 | 7.7 | 8.0 | 4.7 | 6.9 | 7.1 | 4.8 | 8.1 | 9.2 | 7.0 | 7.6 | 7.5 | 6.5 |
| **Age** |  |  |  |  |  |  |  |  |  |  |  |  |  |  |  |
| 24 ≦ | 226 | 6.6 | 7.1 | 6.6 | 6.6 | 4.0 | 8.8 | 6.2 | 5.3 | 11.5 | 7.1 | 8.8 | 8.4 | 8.0 | 4.9 |
| 25 − 29 | 314 | 6.1 | 4.1 | 8.9 | 6.4 | 3.8 | 5.1 | 7.6 | 4.1 | 5.4 | 12.7 | 9.6 | 8.9 | 8.0 | 9.2 |
| 30 − 39 | 239 | 12.1 | 10.9 | 8.8 | 6.7 | 6.3 | 2.5 | 10.0 | 7.9 | 3.8 | 8.4 | 2.9 | 7.1 | 7.5 | 5.0 |
| ≧ 40 | 212 | 9.9 | 5.2 | 5.7 | 13.2 | 5.2 | 12.3 | 3.8 | 1.9 | 13.2 | 7.1 | 5.7 | 5.2 | 6.1 | 5.7 |
| **Clinical Ladder Level** | | |  |  |  |  |  |  |  |  |  |  |  |  |  |
| Level 0 | 155 | 5.2 | 7.1 | 3.9 | 5.8 | 7.1 | 3.9 | 7.1 | 8.4 | 13.5 | 11.0 | 9.0 | 5.2 | 9.0 | 3.9 |
| Level I | 220 | 7.7 | 6.8 | 8.6 | 6.4 | 2.7 | 11.4 | 8.6 | 3.6 | 4.1 | 8.2 | 9.1 | 9.5 | 6.4 | 6.8 |
| Level II | 313 | 6.7 | 5.8 | 11.2 | 11.5 | 5.1 | 4.2 | 10.2 | 2.2 | 8.6 | 10.9 | 1.6 | 3.8 | 10.5 | 7.7 |
| Level III | 303 | 12.5 | 7.3 | 5.3 | 6.6 | 4.6 | 7.9 | 2.6 | 6.6 | 7.6 | 7.3 | 9.9 | 11.2 | 4.3 | 6.3 |
| **Years of Service** | |  |  |  |  |  |  |  |  |  |  |  |  |  |  |
| 0 | 108 | 7.4 | 10.2 | 3.7 | 6.5 | 5.6 | 5.6 | 7.4 | 1.9 | 10.2 | 11.1 | 7.4 | 7.4 | 10.2 | 5.6 |
| 1 − 2 | 268 | 6.3 | 5.6 | 7.1 | 5.2 | 3.0 | 10.4 | 9.3 | 5.2 | 7.1 | 9.7 | 9.7 | 9.3 | 6.3 | 5.6 |
| 3 − 6 | 176 | 8.5 | 3.4 | 2.8 | 13.6 | 5.1 | 9.7 | 0.6 | 5.7 | 12.5 | 8.5 | 8.0 | 6.8 | 8.0 | 6.8 |
| 7 − 12 | 272 | 5.5 | 5.9 | 11.4 | 6.6 | 5.9 | 2.9 | 6.6 | 3.3 | 7.4 | 10.7 | 7.4 | 6.6 | 9.9 | 9.9 |
| ≧ 13 | 167 | 17.4 | 10.8 | 10.2 | 9.6 | 4.8 | 5.4 | 10.8 | 7.8 | 4.8 | 5.4 | 0.6 | 7.2 | 3.0 | 2.4 |
| **Night** |  |  |  |  |  |  |  |  |  |  |  |  |  |  |  |
| **Total** | 1050 | 8.8 | 6.8 | 7.9 | 7.9 | 4.8 | 6.7 | 6.6 | 5.6 | 7.8 | 8.4 | 7.4 | 7.4 | 6.9 | 7.1 |
| **Age** |  |  |  |  |  |  |  |  |  |  |  |  |  |  |  |
| 24 ≦ | 233 | 7.7 | 6.9 | 6.4 | 6.4 | 4.3 | 8.6 | 5.6 | 6.0 | 11.6 | 6.0 | 9.4 | 9.4 | 7.3 | 4.3 |
| 25 − 29 | 329 | 6.4 | 4.6 | 9.4 | 7.0 | 4.9 | 4.3 | 7.3 | 5.2 | 4.9 | 11.9 | 8.5 | 10.0 | 6.7 | 9.1 |
| 30 − 39 | 254 | 12.2 | 9.8 | 9.1 | 6.3 | 5.1 | 2.4 | 10.2 | 8.7 | 4.3 | 8.3 | 4.7 | 5.9 | 7.1 | 5.9 |
| ≧ 40 | 234 | 9.4 | 6.4 | 6.0 | 12.4 | 4.7 | 12.8 | 2.6 | 2.6 | 12.0 | 6.0 | 6.8 | 3.4 | 6.4 | 8.5 |
| **Clinical Ladder Level** | | |  |  |  |  |  |  |  |  |  |  |  |  |  |
| Level 0 | 158 | 6.3 | 7.0 | 3.8 | 5.7 | 6.3 | 4.4 | 6.3 | 9.5 | 13.3 | 9.5 | 8.2 | 6.3 | 9.5 | 3.8 |
| Level I | 232 | 8.6 | 6.9 | 8.6 | 6.5 | 3.0 | 9.5 | 8.2 | 4.7 | 4.3 | 6.9 | 10.3 | 10.8 | 5.6 | 6.0 |
| Level II | 333 | 6.3 | 6.3 | 12.0 | 11.4 | 5.7 | 4.8 | 9.6 | 2.7 | 8.1 | 10.5 | 1.5 | 3.6 | 9.3 | 8.1 |
| Level III | 327 | 12.5 | 7.0 | 5.2 | 6.4 | 4.3 | 7.6 | 2.4 | 7.3 | 7.3 | 6.7 | 11.0 | 9.5 | 4.0 | 8.6 |
| **Years of Service** | |  |  |  |  |  |  |  |  |  |  |  |  |  |  |
| 0 | 109 | 9.2 | 10.1 | 3.7 | 6.4 | 3.7 | 6.4 | 7.3 | 2.8 | 9.2 | 10.1 | 5.5 | 9.2 | 11.0 | 5.5 |
| 1 − 2 | 279 | 7.2 | 5.7 | 7.5 | 5.4 | 3.2 | 9.0 | 8.2 | 6.1 | 7.5 | 8.2 | 11.1 | 10.0 | 5.7 | 5.0 |
| 3 − 6 | 203 | 9.4 | 4.9 | 3.0 | 12.3 | 4.4 | 9.4 | 1.5 | 5.9 | 10.8 | 6.9 | 9.4 | 4.4 | 7.9 | 9.9 |
| 7 − 12 | 282 | 5.7 | 6.0 | 11.3 | 6.7 | 7.1 | 2.8 | 6.4 | 3.9 | 7.1 | 11.3 | 6.4 | 7.1 | 8.2 | 9.9 |
| ≧ 13 | 177 | 15.3 | 9.6 | 11.3 | 9.6 | 4.5 | 6.2 | 9.6 | 9.0 | 5.1 | 4.5 | 2.3 | 6.2 | 2.8 | 4.0 |

#### **Distribution of the Number of Records per Nurse**


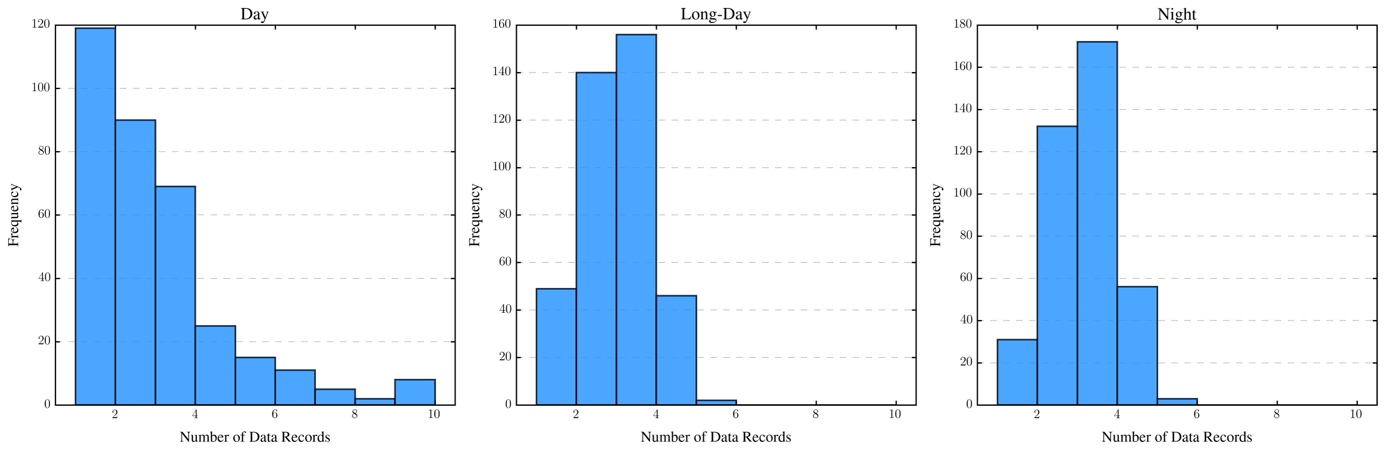


Fig. 10: Histograms of the number of records per nurse for each shift

Figure 10 shows the histograms of the number of records per nurse, separately for day, long-day, and night shifts, where most nurses contributed between one and five observations per shift type, and the distribution is reasonably concentrated. Although, in day shift, the exact number of data records varied slightly across individuals, the spread was not extreme, and no nurse disproportionately influenced the dataset. This slight variation in the day shift is partly attributable to the presence of nurses who do not work night shifts and therefore appear only in day-shift records.

#### **Normality and Homoscedasticity**


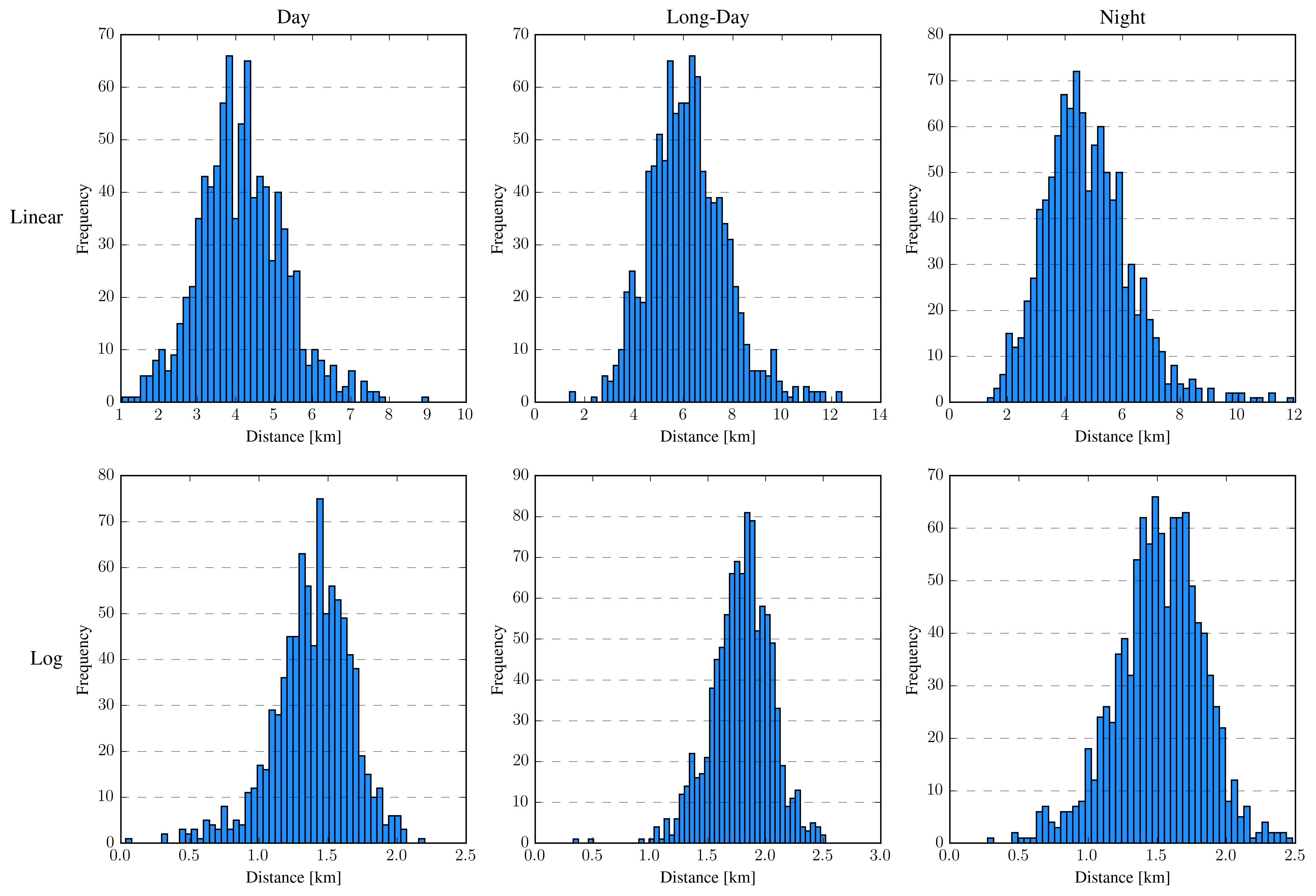


Fig. 11: Histograms for each shift on normal and log scale


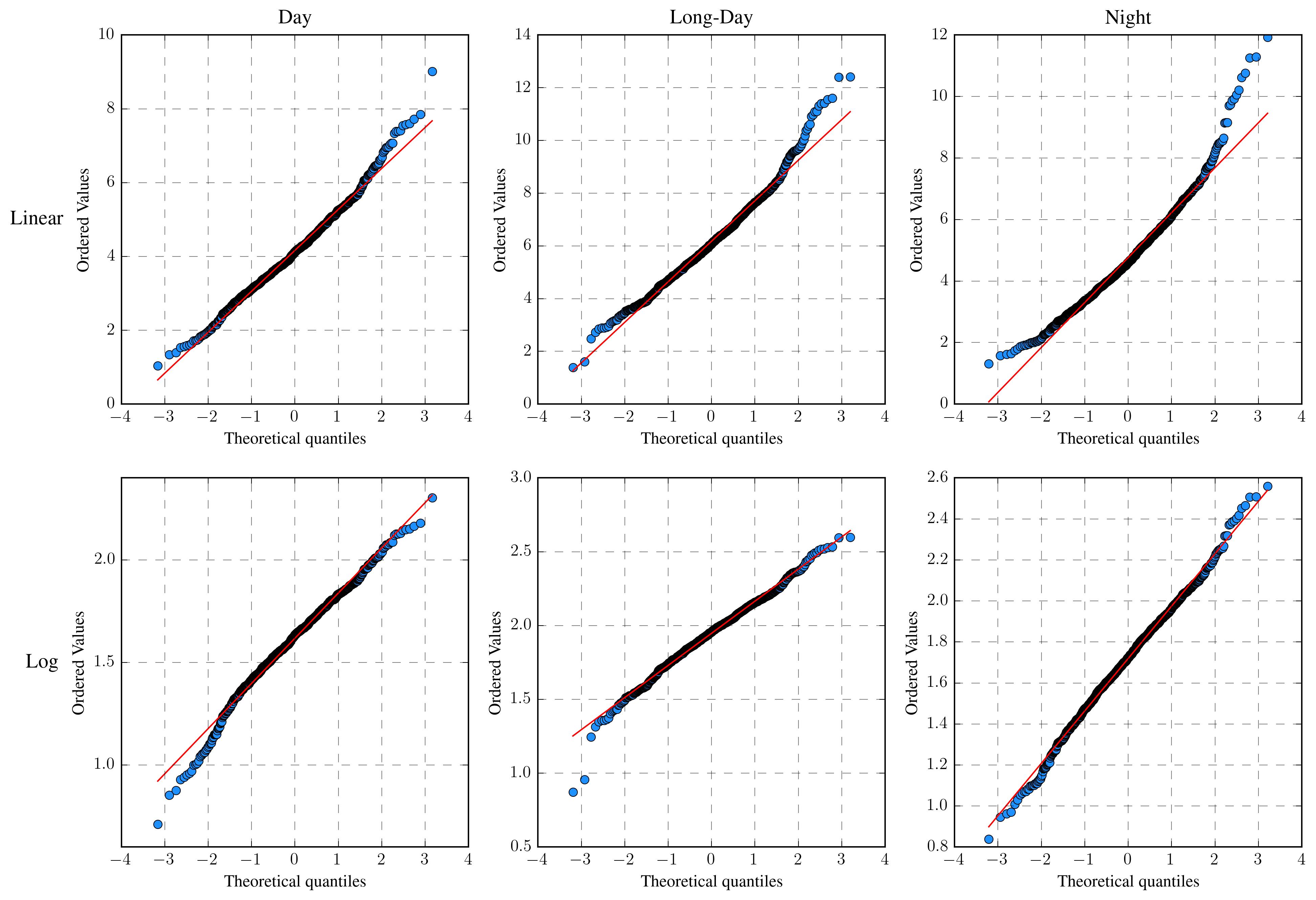


Fig. 12: QQ-plots for each shift on normal and log scale

Figures 11 and 12 show the histograms and QQ-plots of nurses’ walking distance on normal and log scales for each shift. We used the Shapiro-Wilk test [1, 2] to test for normality. The p-values for day, long-day, and night shifts on the normal scale were $9.72\times{10}^{-6}$, $1.76\times{10}^{-9}$, and $1.88\times{10}^{-14}$, respectively, while those on the log scale were $2.72\times{10}^{-11}$, $2.24\times{10}^{-9}$, and $5.31\times{10}^{-6}$, respectively. These results indicate that the distribution of the data is neither normal nor lognormal.

Given the non-normal distribution of walking distances, we applied Levene’s test [3] to check for homoscedasticity across groups. The p-values for the following groups were less than $0.05$: clinical ladder level and age for long-day shifts and clinical ladder level, age, and years of service for night shifts, indicating heteroscedasticity.

#### **Variance Inflation Factor**

We calculated the variance inflation factor (VIF) to check for multicollinearity. Due to high VIF values, i.e., more than $50$, we removed gender, years of service, occupancy rate, bed utilization rate, and the number of nurses. The final VIF results for each shift are shown in Table 8. Although VIF values (bold) for age, general calls, and PNR were greater than $10$, these variables were retained due to their importance in the analysis.

Table 8: Final VIF results for each shift

| Variables | Day | Long-Day | Night |
| --- | --- | --- | --- |
| Age | **22.4** | **24.6** | **25.2** |
| Level 0 | 2.5 | 1.8 | 1.7 |
| Level II | 3.4 | 2.8 | 2.8 |
| Level III | 4.7 | 4.6 | 4.7 |
| General Calls | **13.3** | **13.2** | 8.4 |
| Sensor Calls | 5.2 | 5.0 | 4.2 |
| PNR | **31.5** | **27.7** | **24.0** |
| Weekend | 2.6 | 1.5 | 1.4 |
| Ward A | 1.7 | 1.9 | 2.0 |
| Ward B | 3.6 | 3.5 | 4.1 |
| Ward C | 1.3 | 1.5 | 1.6 |
| Ward E | 1.9 | 1.9 | 1.8 |
| Ward F | 2.1 | 2.2 | 2.0 |
| Ward H | 1.4 | 1.3 | 1.4 |
| Ward I | 1.9 | 2.3 | 2.0 |
| Ward J | 1.9 | 2.3 | 2.3 |
| Ward K | 2.8 | 2.4 | 2.2 |
| Ward L | 1.9 | 1.9 | 1.9 |
| Ward M | 2.3 | 2.0 | 1.9 |
| Ward N | 2.3 | 2.7 | 3.2 |

**References**

[1] S. S. Shapiro and M. B. Wilk, “An Analysis of Variance Test for Normality (Complete Samples),” *Biometrika*, vol. 52, no. 3–4, pp. 591–611, 1965.

[2] N. M. Razali and Y. B. Wah, “Power Comparisons of Shapiro-Wilk, Kolmogorov-Smirnov, Lilliefors and Anderson-Darling Tests,” *Journal of Statistical Modeling and Analytics*, vol. 2, no. 1, pp. 21–33, 2011.

[3] M. B. Brown and A. B. Forsythe, “Robust Tests for the Equality of Variances,” *Journal of the American Statistical Association*, vol. 69, no. 346, pp. 364–367, 1974.
